# De novo genome hybrid assembly and annotation of the endangered and euryhaline fish *Aphanius iberus* (Valenciennes, 1846) with identification of genes potentially involved in salinity adaptation

**DOI:** 10.1186/s12864-025-11327-0

**Published:** 2025-02-12

**Authors:** Alfonso López-Solano, Ignacio Doadrio, Tessa Lynn Nester, Silvia Perea

**Affiliations:** 1 Museo Nacional de Ciencias Naturales, C/ José Gutiérrez Abascal, 2, 28006 Madrid, Spain; 2 Tragsatec. Grupo Tragsa, C/ Julián Camarillo 6B, Madrid, 28037 Spain

**Keywords:** Reference genome, *Aphanius iberus*, Cyprinodontiformes, De novo hybrid assembly, Annotation

## Abstract

**Background:**

The sequencing of non-model species has increased exponentially in recent years, largely due to the advent of novel sequencing technologies. In this study, we construct the Reference Genome of the Spanish toothcarp (*Aphanius iberus* (Valenciennes, 1846)), a renowned euryhaline fish species. This species is native to the marshes along the Mediterranean coast of Spain and has been threatened with extinction as a result of habitat modification caused by urbanization, agriculture, and its popularity among aquarium hobbyists since the mid-twentieth century. It is also one of the first Reference Genome for Euro-Asian species within the globally distributed order Cyprinodontiformes. Additionally, this effort aims to enhance our comprehension of the species' evolutionary ecology and history, particularly its remarkable adaptations that enable it to thrive in diverse and constantly changing inland aquatic environments.

**Results:**

A hybrid assembly approach was employed, integrating PacBio long-read sequencing with Illumina short-read data. In addition to the assembly, an extensive functional annotation of the genome is provided by using AUGUSTUS, and two different approaches (InterProScan and Sma3s). The genome size (1.15 Gb) is consistent with that of the most closely related species, and its quality and completeness, as assessed with various methods, exceeded the suggested minimum thresholds, thus confirming the robustness of the assembly. When conducting an orthology analysis, it was observed that nearly all genes were grouped in orthogroups that included genes of genetically similar species. GO Term annotation revealed, among others, categories related with salinity regulation processes (ion transport, transmembrane transport, membrane related terms or calcium ion binding).

**Conclusions:**

The integration of genomic data with predicted genes presents future research opportunities across multiple disciplines, such as physiology, reproduction, disease, and opens up new avenues for future studies in comparative genomic studies. Of particular interest is the investigation of genes potentially associated with salinity adaptation, as identified in this study. Overall, this study contributes to the growing database of Reference Genomes, provides valuable information that enhances the knowledge within the order Cyprinodontiformes, and aids in improving the conservation status of threatened species by facilitating a better understanding of their behavior in nature and optimizing resource allocation towards their preservation.

**Supplementary Information:**

The online version contains supplementary material available at 10.1186/s12864-025-11327-0.

## Background

Recent advances in genomics have created an unprecedented opportunity to resolve long-standing questions regarding the evolutionary processes of organisms. These questions were previously difficult to understand without comprehensive genomic analyses. Specifically, it is particularly interesting the study of unusual adaptations in species which thrive in dynamic and ever-changing habitats, such as the genetic basis of fish adaptations, such as responses to hypoxia and air exposure, and fishes facing fluctuating salinity levels in brackish waters [1–3]. In light of the ongoing global warming that has resulted in significant and rapid environmental transformations, this kind of investigation has become increasingly relevant. Aquatic ecosystems, especially those in inland environments, are particularly vulnerable to the adverse effects of climate change and face immense threats, largely due to anthropogenic pressures that have significantly altered and transformed them [4]. Climate change in the Mediterranean region is increasing the frequency and intensity of catastrophic and unpredictable events, such as cut-off low storms (DANAs) and droughts.

Fish populations are especially susceptible to the consequences of climate change, including rising average temperatures, alterations in biological oxygen demand, and greater fluctuations in salinity. These challenges are particularly significant for euryhaline fish species, renowned for their remarkable adaptability to extensive salinity and temperature ranges. For instance, these species possess intricate physiological mechanisms that enable them to efficiently regulate their osmotic balance in response to fluctuations in water salinity [5–12]. Nonetheless, alterations in salinity patterns triggered by climate change disrupt the delicate equilibrium that is essential for the well-being of euryhaline fishes [13]. This underscores the need for a more comprehensive understanding of the functioning of various adaptation mechanisms that different species have evolved to survive in these highly distinctive environments, as well as the enhancement of our understanding of their stress resistance [1, 3]. Additionally, numerous fish species that inhabit coastal lagoons, salty rivers, and other coastal water bodies are experiencing drastic population declines, posing a severe threat to their survival [14–17].

**Fig. 1:**
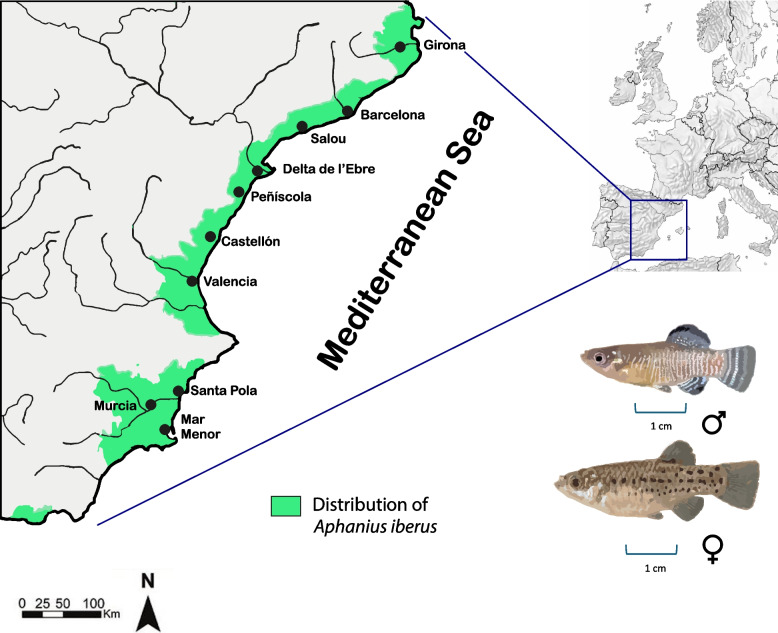
Current distribution of the Spanish toothcarp (*Aphanius iberus*) along the eastern coast of the Iberian Peninsula. Bottom right are a male (top) and a female (bottom) individuals of *A. iberus*

The Spanish toothcarp, *Aphanius iberus* (Valenciennes, 1846), is one of those singular species that has evolved unique traits enabling it to thrive in highly variable, dynamic, and demanding habitats along the eastern coast of Spain, including groundwater springs (locally known as ullals), coastal lagoons, river mouths, and even salt marshes [18] (Fig. 1). The species is classified as "Endangered" (IUCN category: EN; National and European legislation—Habitats Directive of the Council of Europe, Act 1992; National Catalog of Threatened Species, Act 2011) due to various factors, including pollution, habitat destruction, unregulated management by aquarium enthusiasts, and the presence of invasive species, which are displacing it from its natural environment to more hostile habitats [18–20]. Intensive management programs, such as habitat restoration and stocking, are in place to protect the species [21, 22]. Despite its conservation status and remarkable adaptability, the Spanish toothcarp has not been recognized as a model species for genetic studies. Nevertheless, numerous investigations have been conducted to explore its genetic structure and diversity using traditional methods such as allozymes, mitochondrial genes and microsatellites [19, 23–26], alongside contemporary Next-generation sequencing methods [27, 28]. Analyses of the species genetic structure based on different markers have been conducted in particular within the context of conservation [29–31]. Nevertheless, evolutionary questions regarding its remarkable adaptability to the variable and changing environmental conditions in which the species naturally inhabits remain unanswered. One potential avenue for addressing this knowledge gap is through the analysis of loci under selection in a broad genome context to understand how populations response to environmental changes in the ongoing global warming. In the meantime, analogous genetic studies have been conducted in other fish species with the objective of deepening our understanding of their biology and expanding our knowledge of comparative genomics, adaptations genomics, and functional gene variation [28, 32–35], among others. Subsequently, there has been a notable increase in the number of publications related to Reference Genomes. The total number of Reference Genomes available on GenBank in the order Cyprinodontiformes has grown exponentially. The findings of these studies can be applied to *A. iberus*, a species with an exceptional capacity for adaptation to fluctuating saline environments, and other limiting environmental conditions, in a wider evolutionary context within the Cyprinodontiformes order underscoring the necessity for a Reference Genome of the species.

In the present study, a high-quality genome assembly of *A. iberus* was generated for the first time using a de novo hybrid assembly strategy that combined both high-coverage Pacific Biosciences (PacBio) long-read sequencing with precise Illumina short-read data. The strategy of combining two or more sequencing techniques has significantly increased the availability of Reference Genomes, primarily driven by the substantial advancements in the accuracy and cost-effectiveness of genome sequencing [36, 37]. Furthermore, gene prediction was performed using AUGUSTUS (3.2.3) [38] and, in order to improve robustness, two different approaches, InterProScan v5.50–84.0 [39] and Sma3s v2 [40], were utilized for functional annotation of the genes. Additionally, various analyses, including orthologous comparisons and the construction of a phylogenetic tree, were performed to compare the newly sequenced genome with previously published and well-annotated genomes of closely related genera. The fully sequenced and annotated genome of the Spanish toothcarp, the first Euro-Asian species of the order Cyprinodontiformes to be released, provides an invaluable genetic resource for investigating the mechanisms of evolutionary adaptation in these species, which may be linked to the dynamic history of the coastline in this region, as well as facilitating future studies in ecology, phylogenetics or evolution.

## Methods

### DNA extraction

DNA was extracted from muscle tissue from three samples collected at the Centro de Conservación de Especies Dulceacuícolas which belongs to the Government of Valencia (Spain) ("Piscifactoría de El Palmar"), Valencia, Spain, serving as a genetic refuge for various populations of the Spanish toothcarp. The three specimens come from the Albuixech population, which represents the most widely distributed genetic lineage of the Spanish toothcarp [19, 24, 29]. The fishes were euthanized using 0.1% tricaine methanesulfonate (MS222) following the standard internationally approved protocols by qualified personal at the mentioned center in Valencia and posteriorly sent to the National Museum of Natural Sciences in Madrid. DNA isolation was performed using the MagAttract HMW DNA isolation kit (Qiagen) and the final elution step was carried out with a volume of 100 μL. DNA quantification was performed using the Qubit High Sensitivity dsDNA Assay (Thermo Fisher Scientific).

### Library preparation and genome assembly

We opted for sequencing the data from PacBio and Illumina DNA sequencing in order to obtain trustworthy assembly and annotation. Prior to library preparation, the sample was further purified and size-selected to keep the largest fragments.

### PacBio and Illumina library preparation and sequencing

For library preparation, the SMRTbell Express Template Prep Kit 2.0 (PacBio) was utilized in accordance with the manufacturer's instructions. Subsequently, sequencing was performed on a Sequel II sequencer (PacBio) with a SMRT Cell 8M, using the Long-reads mode. The Illumina DNA Prep kit was used for preparing the Illumina library in strict accordance with the manufacturer's guidelines. The Agilent 2100 Bioanalyzer was used to verify the library's fragment size distribution and concentration using the Agilent HS DNA Kit. Then, the library was sequenced on a portion of a NovaSeq PE150 flow cell with a total output target of 50 Gb.

### Raw data pre-processing

The *de novo* genome sequencing using the Illumina NovaSeq platform generated a total of 436,121,806 paired-end reads (R1+R2). The raw fastq files underwent quality assessment using the software FastQC v0.11.5 [41]. In addition, 7,307,982 subreads were obtained by *de novo* genome sequencing using the PacBio Sequel II platform. The PacBio reads were quality-checked using SequelTools software [42], giving the longest subread of 50.93Mbp with a mean read length of 9,349bp and a N50 value of 13,224bp (see Supplementary Table 1).

### De novo hybrid assembly

Two distinct *de novo* approaches were utilized to assemble the genome of *A. iberus.* Firstly, the short and long genomic sequencing reads were assembled de novo into “mega-reads” using the software MaSuRCA v3.4.2 [43], which incorporates the advantages of combining de Bruijn graph and Overlap-Layout-Consensus assembly approaches. QuorUM software [44] was used following the instructions, to error-correct the short reads, which were then extended into "super-reads" [45] and aligned to the long reads. Consistent alignments of "super-reads" were merged into "mega-reads," and Flye v2.5 [46], implemented in MaSuRCA, was used to assemble the "mega-reads”. For the second approach, it was used the software HASLR [47]. This software uses a new data structure called a backbone graph in addition to the de Bruijn graph and the SPOA algorithm.

After generating the assemblies, both MaSuRCA and HASLR were polished using POLCA [48], which involved aligning the raw Illumina reads to the assembly using the BWA mem algorithm [49], and calling short variants using FreeBayes [50]. Subsequently, the quality of the assemblies, both before and after polishing, was assessed using QUAST 5.0.2 [51]. The number of scaffolds obtained were 3,026 for MaSuRCA polished and 8,313 for HASLR polished. The total length of the genome assembly was 1,198,861,738bp and 1,131,748,719bp respectively for each method (Supplementary Table 2). The Scaffold N50 and L50 were 1,678,775bp and 201 for MaSuRCA polished and 284,695bp and 1,180 for HASLR polished. The GC content was 39.17% and 39.12% respectively after each polishing process.

The software KMC ver. 3.1.1 [52] was employed to enumerate the frequency of *k-mers* in the corrected reads, with the *k-mer* size parameter set to k=21. This analysis helps to detect sequencing errors, contamination, or repetitive sequences, and aids in determining whether the genome assembly process was successful. The resulting *k-mer* profile was generated using GenomeScope 2.0 [53] and is depicted in Supplementary Figure 1.

### Quality control of the hybrid assembly

The genomic coverage of each region was determined by mapping short sequencing reads to the genome assembly produced by MaSuRCA, using BWA v0.7.15 [54], and the mapping statistics were calculated with SAMtools 1.3.1 [55].

**Fig. 2:**
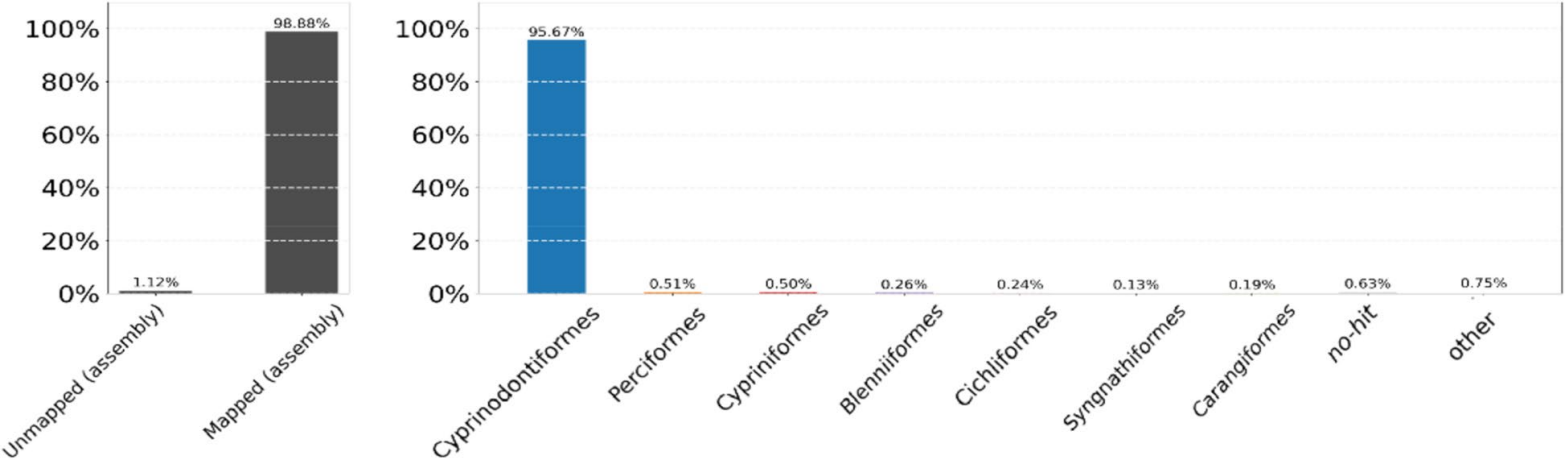
ReadCovPlot of the *A. iberus* assembly, displaying on the left the proportion of mapped and unmapped reads against the assembly, and on the right the percentage of mapped reads assigned to different taxa at the rank of 'order'

To identify potential contaminant sequences in the assembly, BlobTools v1.1.1 was used to create a scatter plot and a bar chart (see Figure 2 and Supplementary Figure 2). The scatter plot represents assembly contigs/scaffolds as dots colored according to their taxonomic affiliation, based on sequence similarity search results. The bar chart illustrates the sequences mapped and unmapped against the assembly and mapped sequences assigned to different taxa.

The completeness and quality of the genome assembly were assessed using the "genome" mode of BUSCO V5.5 (Benchmarking Universal Single-Copy Orthologs) V5.beta.1 [56]. To assess the results of *A. iberus* and to make comparisons between species, BUSCO analysis was conducted on three closely related species: *Cyprinodon variegatus* (NCBI GenBank: GCA_000732505.1)*, Poecilia formosa* (NCBI GenBank: GCA_000485575.1) and *Xiphophorus maculatus* (NCBI GenBank: GCA_002775205.2). To predict eukaryotic genes, the Metaeuk pipeline [57] was utilized with default parameters and the lineage-specific cyprinodontiformes_odb10 database (last update 2021-02-19) [56].

To conduct a comprehensive genome-wide comparison, we utilized *Poecilia reticulata,* a closely related species with a chromosome-level well-characterized genome. The complete Reference Genome sequence for *P. reticulata* was obtained from NCBI GenBank (GCF_000633615.1) and aligned against the newly assembled genome of *A iberus* using minimap2 [58]. This alignment served as input for D-GENIES [59] in order to compare similarity between both genomes.

### Repetitive elements

De novo identification of repetitive elements in the assembly was performed using RepeatModeler v2.0.1 [60]. The assembly was then masked using RepeatMasker v4.1.2-p1 [61] and the Repbase-20170127 library of known repeats [62]. The number and length of masked repeats, classified by repeat class, are reported in Supplementary Table 3.

### Genome annotation

The first step in genome annotation in a given genomic sequence is to predict all gene structures. Gene prediction was conducted using AUGUSTUS v.3.2.3 [38] which defines probability distributions for the different sections of genomic sequences (i.e. exons, introns, intergenic regions) based on a generalized Hidden Markov Model. *Danio rerio* was the reference species used as a setting. The result obtained was a gff (General Feature Format) which was employed as an input for the utility gffread v0.12.6 [63]. This utility extracts the sequence of all transfrags, which are transcripts or fragments that result from the assembly process, generating a FASTA file with all the predicted sequences. To identify candidate coding regions within these predicted mRNA sequences, TransDecoder v5.5.0 [64] was used. This software is particularly useful in the analysis of incomplete genomes or in the identification of new genes in non-model or understudied species.

To gain the maximum possible information about the biological function of the predicted genes, two different approaches were performed to functionally annotate them:

The precited protein-coding genes were initially functionally annotated using the software InterProScan v5.50–84.0 [39]. The annotation was performed using general-content databases, including: the conserved domain database (CDD) [65], the Coils database [66], the Gene3D database [67], the HAMAP database [68], the MobiDBLite database [69], the protein analysis through evolutionary relationships (PANTHER) classification system [70], the protein families database (Pfam) [71], the protein information resource and superfamily (PIRSF) classification system [72], the protein motif fingerprints (PRINTS) database [73], the protein domains, families and functional sites (ProSitePatterns and ProSiteProfiles) databases [74], the structure function linkage (SFLD) database [75], the simple modular architecture research tools (SMART) [76], the SUPERFAMILY database [77], and the TIGRFAM database [78].

The second annotation was performed using the software Sma3s v2 [40], and the complete manually annotated and reviewed Swiss-Prot database from UniProtKB [79]. Sma3s reports a summary with different categories and the number of sequences belonging to each functional category. Sequence annotations also contain the most probable gene name and the most probable description (including putative EC enzyme codes).

Gene Ontology (GO) Terms were generated for both annotation methods, and genes were classified into three categories, GO Function, which includes the genes in general categories of Molecular Function; GO Process, which includes genes in different categories of Biological Processes; and GO Component, which includes genes in different categories of Cellular Components. Many genes were classified in more than one category due to its multifunctionality.

The information obtained from the performed annotations is compiled in a table accessible through DIGITAL.CSIC: "Annotation_Aphanius_iberus.xslx" (http://hdl.handle.net/10261/365271). The table includes 73,242 predicted genes, with each gene's location within a Scaffold, its length, strand orientation (+ or -), mRNA sequence, and all annotation features conducted with InterProScan and Sma3s, as described previously.

### Phylogenomics and comparative genomics

A set of fourteen species from various freshwater fish genera representative of different orders, for which Reference Genome data were available on NCBI GenBank, were selected to conduct different analyses, with a particular focus on the Cyprinodontiformes genera. Afterwards, their complete protein sequences were downloaded from the database (Table 1). The most closely related species was *Cyprinodon variegatus*, from the same order as *A. iberus*. Four other species also belonged to the same order and five more to the same superorder. Predominantly, most species were phylogenetically closely related, however, some distantly related ones were also included in the analysis due to their extensive use as model species in several studies [80–87].

**Table 1:** Genome size comparison to some related species to *A. iberus*

Species	Order	Family	Genome size (Mb)	Accesion number
*Aphanius iberus* (Valenciennes, 1846)	Cyprinodontiformes	Aphaniidae	1,199	GCA_028564705.1
*Cyprinodon variegatus* (Lacepède, 1803)	Cyprinodontiformes	Cyprinodontidae	1,035	GCA_000732505.1
*Xiphophorus maculatus* (Günther, 1866)	Cyprinodontiformes	Poeciliidae	704.3	GCA_002775205.2
*Gambusia affinis* (Baird and Girard, 1853)	Cyprinodontiformes	Poeciliidae	680.1	GCA_019740435.1
*Poecilia formosa* (Girard, 1859)	Cyprinodontiformes	Poeciliidae	748.9	GCA_000485575.1
*Fundulus heteroclitus* (Linnaeus, 1766)	Cyprinodontiformes	Fundulidae	1,203	GCA_011125445.2
*Oryzias latipes* (Temminck and Schlegel, 1846)	Beloniformes	Adrianichthyidae	734	GCA_002234675.1
*Gasterosteus aculeatus* (Linnaeus, 1758)	Gasterosteiformes	Gasterosteidae	471.9	GCA_016920845.1
*Oreochromis niloticus* (Linnaeus, 1758)	Cichliformes	Cichlidae	1,006	GCA_001858045.3
*Takifugu rubripes* (Temminck and Schlegel, 1850)	Tetraodontiformes	Tetraodontidae	384.1	GCA_901000725.2
*Danio rerio* (Hamilton-Buchanan, 1822)	Cypriniformes	Danionidae	1,373	GCA_000002035.4
*Cyprinus carpio* (Linnaeus, 1758)	Cypriniformes	Cyprinidae	1,680	GCA_018340385.1
*Astyanax mexicanus* (De Filippi, 1853)	Characiformes	Characidae	1,373	GCA_023375975.1
*Gadus morhua* (Linnaeus, 1758)	Gadiformes	Gadidae	669.9	GCA_902167405.1

The downloaded protein sequences were aligned using MAFFT/7.475-with-extension [88], where the newly sequenced data from *A. iberus* were also included. Subsequently, TrimAl was employed to evaluate and remove poorly aligned regions [89]. The resulting alignment based on the longest isoform was used as input for the identification of orthologous genes in the studied species. We employed OrthoFinder 2.5.4 [90], to cluster homologous genes from all 14 species through sequence similarity [90], among other functionalities.

A phylogenetic analysis was conducted with all 14 fish species using the orthogroups clustered by Orthofinder. The phylogenetic tree was inferred based on the single-copy orthologous gene sequences implemented in the IQ-tree software [91], with the LG+F+I+G4 substitution model for proteins, as estimated by ModelFinder implemented in IQ-tree [92, 93]. The amino acid sequences were concatenated and treated as a single partition, which is the default setting of ModelFinder. The tree was rooted using a midpoint root approximation and branch support was evaluated with 1000 bootstrap replicates based on the ultrafast algorithm [94].

To assess more accurately the overlap in orthologous genes between *A. iberus* and the rest of species, a new analysis using Orthofinder was performed. This analysis specifically incorporated only two cyprinodontiform closely related species of *A. iberus* (Valenciennes, 1846): Sheepshead minnow *Cyprinodon variegatus* (Lacepède, 1803)*,* and the Amazon molly *Poecilia formosa* (Girard, 1859) along with one species of a closely related genus from its sister order Beloniformes: the Japanese medaka *Oryzias latipes* (Temminck y Schlegel, 1846).

## Results

High molecular weight (HMW) DNA was extracted from muscle tissue from three individual samples collected at the Centro de Conservación de Especies Dulceacuícolas, belonging to the Government of Valencia ("Piscifactoría de El Palmar"), Valencia, Spain. We pursued a de novo hybrid assembly strategy employing long-read PacBio and short-read Illumina DNA sequencing, along with two distinct assembly approaches in order to obtain a trustworthy assembly and annotation (See Material & Methods section). The de novo genome sequencing in the PacBio Sequel II platform yielded a total of 7.3 million subreads (mean read length 9.6 kbp; N50 13,224). The de novo genome sequencing in the Illumina NovaSeq platform rendered a total of close to 436 million paired-end reads. After assembly, the genome size was estimated to be 1.15 Gb at 95 × coverage (See Supplementary Table 1 for details in assembly quality). Detailed information, including the total length and assembly statistics of this hybrid genome, can be found in Table 2 and Supplementary Table 3.

**Table 2:** General statistics of the hybrid assembly

**Assembly global statistics**	
Total sequence length (bp)	1,198,861,116 bp
Total ungapped length (bp)	1,198,858,230 bp
Gaps between scaffolds	0
Number of scaffolds	3,025
Scaffold N50	1,678,775 bp
Scaffold L50	201
Contig N50	1,625,559 bp
Contig L50	205
GC percent	39%

Different analyses indicated the high accuracy and robustness of the genome assembly and annotation. The plot profile illustrating the observed *k-mer* frequency distribution, based on a *k-mer* size of 21 (the recommended size) in the corrected reads, is presented in Supplementary Fig. 1. The genomic coverage, determined by mapping short sequencing reads to the genome assembly produced by MaSuRCA, resulted in a mapping rate of 98.8% of the read sequences (see Fig. 2 and Supplementary Fig. 1). Furthermore, when SAMtools was employed, the percentage increased to 99.67% of reads mapped back to the assembly. Additionally, Fig. 2 illustrates the percentage of mapped sequences assigned to different taxa, with 95.67% of the sequences belonging to the order Cyprinodontiformes, which indicates a robust affinity of the assembled genomic data with its corresponding taxonomic group (Fig. 2 on the right).

**Fig. 3:**
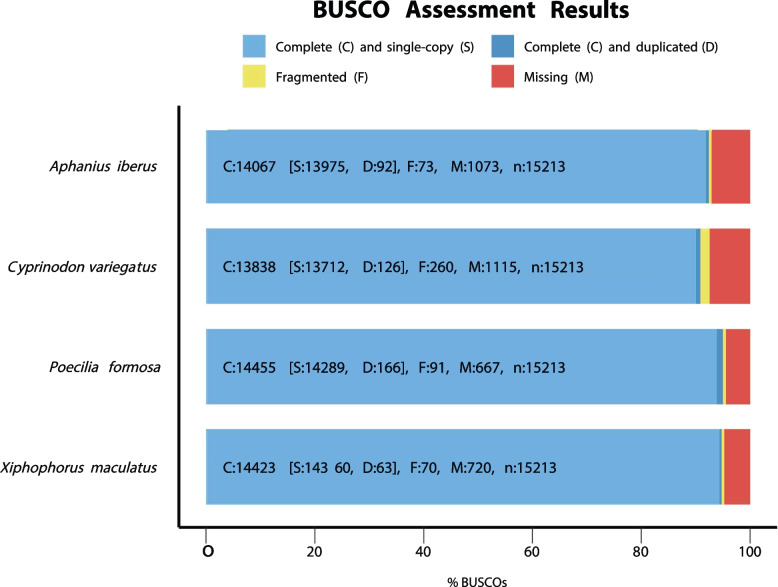
Summary of the BUSCO analysis results for the *A. iberus* assembly and the lineage dataset cyprinodontiformes_odb10 (created 2021–02–19) in comparison with three closely related species

In a comparison of *A. iberus* with three other closely related species in the order Cyprinodontiformes (*Cyprinodon variegatus, Poecilia formosa, and Xiphophorus maculatus*; Table 1), a total of 15,213 BUSCO data sets (n) were analyzed. The results indicated that *A. iberus* exhibited 14,067 complete orthologue genes (C: 92.5%) versus 13,838, 14,455 and 14,423 respectively in the other three species. Of these, 13,975 were single-copy orthologue genes (S: 99.3%), while 92 were duplicated (D: 0.7%) versus 13,712 and 92, 14,289 and 166 and 14,360 and 63 in that order for the other three species. Furthermore, only 0.5% of the remaining orthologue genes were fragmented (F, 73), while approximately 7% were missing (M, 1,073) versus 260 (F) and 1,115 (M) for *C. variegatus*, 91 (F) and 667 (M) for *P. formosa* and 70 (F) and 720 (M) for *X. maculatus* (Fig. 3).

**Fig. 4:**
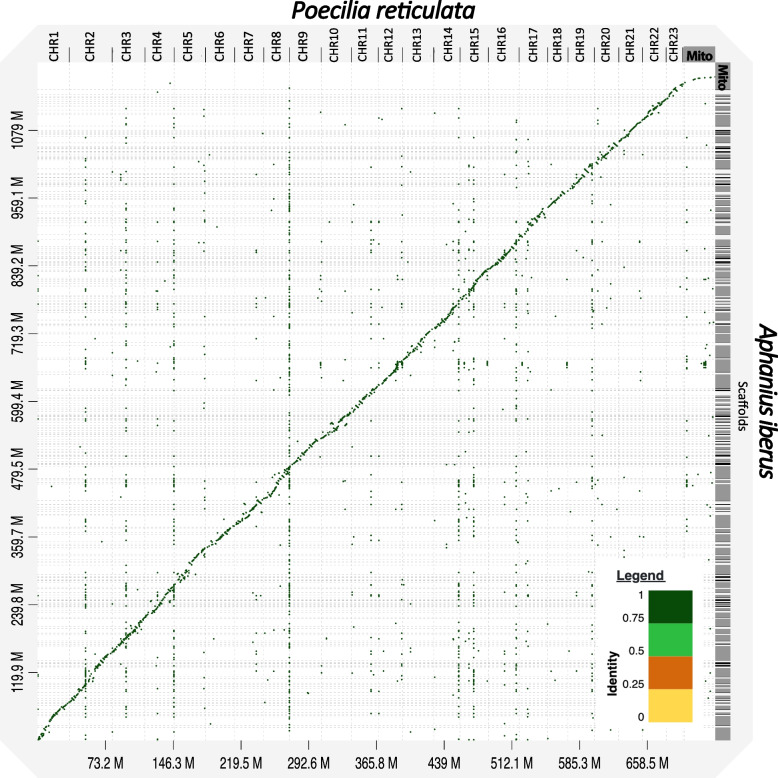
Dot-plot produced using D-Genies for the comparison of *Poecilia reticulata* chromosome-level assembly (horizontal axis) versus *Aphanius iberus* Scaffold-level assembly (vertical axis). Dotted gridlines represent scaffold/chromosome boundaries. Only sequence similarity higher than 0.75 (darker green dots) are represent in the graphics

To conduct a comprehensive genome-wide comparison, a closely related species with an extensively characterized genome, including chromosome-level sequencing, was selected: *Poecilia reticulata* (NCBI GenBank: GCF_000633615.1), that was aligned against the newly assembled genome of *A. iberus* and used as an input for D-GENIES [59]. The results based on a 0.75 identity level suggest that these two genomes exhibited high whole-genome scale similarity (Fig. 4).

Approximately half of the genome (49.10%) was composed of repetitive elements. Among these, DNA transposons were the most abundant, comprising for 16.37% of the genome. Tc1-IS630-Pogo was the most predominant DNA transposon (7.22%) (Table 3, Supplementary Table 3). Retroelements accounted for 14.11% of the genome, with L2/CR1/Rex being the most abundant retroelement within the LINE class, representing a 7.62% of the total abundance. A total of 18.63% of the Repetitive Elements remained unclassified.

**Table 3:** Repetitive elements statistics in percentage and the annotation statistics of the newly sequenced genome of *A. iberus*

Repetitive elements	
Total (%)	49.10
Retroelements (%)	14.11
SINEs (%)	0.41
LINEs (%)	11.26
LTR elements (%)	2.45
DNA transposons (%)	16.37
Unclassified (%)	18.63
**Annotation**	
Predicted genes	73,242
Annotated genes	42,045
Mean [median] gene length (bp)	13,532.7 bp [8,018 bp]
Mean [median] exon length (bp)	195.9 bp [128 bp]
Mean [median] intron length (bp)	1,735.3 bp [541 bp]
Mean [median] exons per gene	7.9 [5]
Mean [median] introns per gene	6.9 [4]

Subsequently, gene prediction yielded a total of 73,242 genes (Table 3). Of the total number of genes, 57.41% (42,045 out of 73,242) of them were functionally annotated using either of the two methods (http://hdl.handle.net/10261/365271 to see the whole annotation). Out of the 19,960 genes annotated with Sma3s, at least 2,300 have been associated with salinity.

**Fig. 5:**
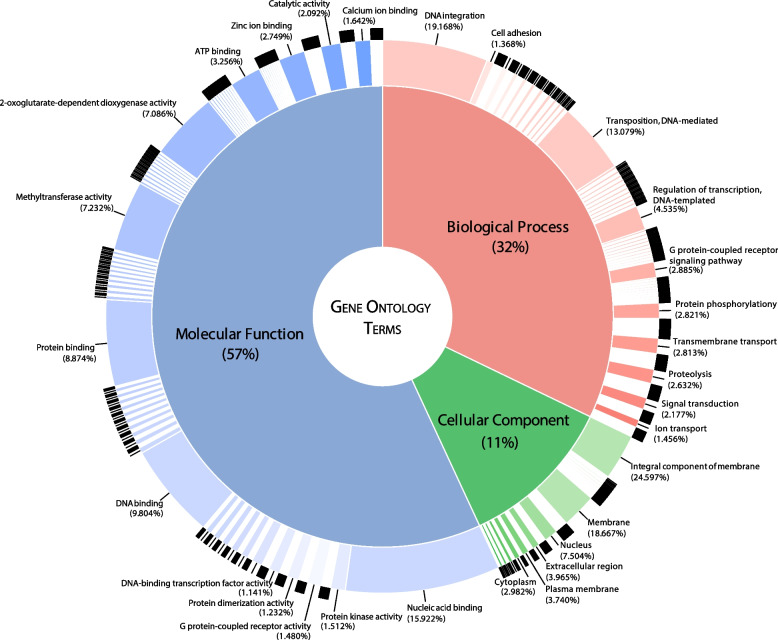
Gene Ontology Terms Treemap. Top GO Molecular Functions in blue, top Biological Processes in red and top Cellular Components in green. The higher % for the different GO terms in each category are represented in the Treemap. Nucleid acid binding and DNA binding are the most abundant GO terms in Molecular Function; DNA integration in Biological Processes and Integral component of membrane in the corresponding Cellular Component

A total of 1,399, 1,629, and 455 Gene Ontology (GO) Terms were identified for Molecular Function, Biological Processes, and Cellular Component, respectively. The most abundant GO Terms related to Molecular Function encompassed Nucleic acid binding, DNA binding and Protein binding. With regard to Biological Processes, the most prevalent GO Terms were DNA integration, Transposition, DNA-mediated and Regulation of transcription, DNA-templated. In the case of Cellular Component, the most common GO Terms were Internal component of membrane, Membrane and Nucleus (Fig. 5 represents GO Terms for each of the three GO categories, with the most abundant Terms for each category appearing with their percentage). The analysis of GO Terms suggested that the majority of annotated genes are primarily associated with DNA processes of duplication, transcription to RNA, and protein synthesis, as well as the movement of these molecules across membranes.

**Fig. 6:**
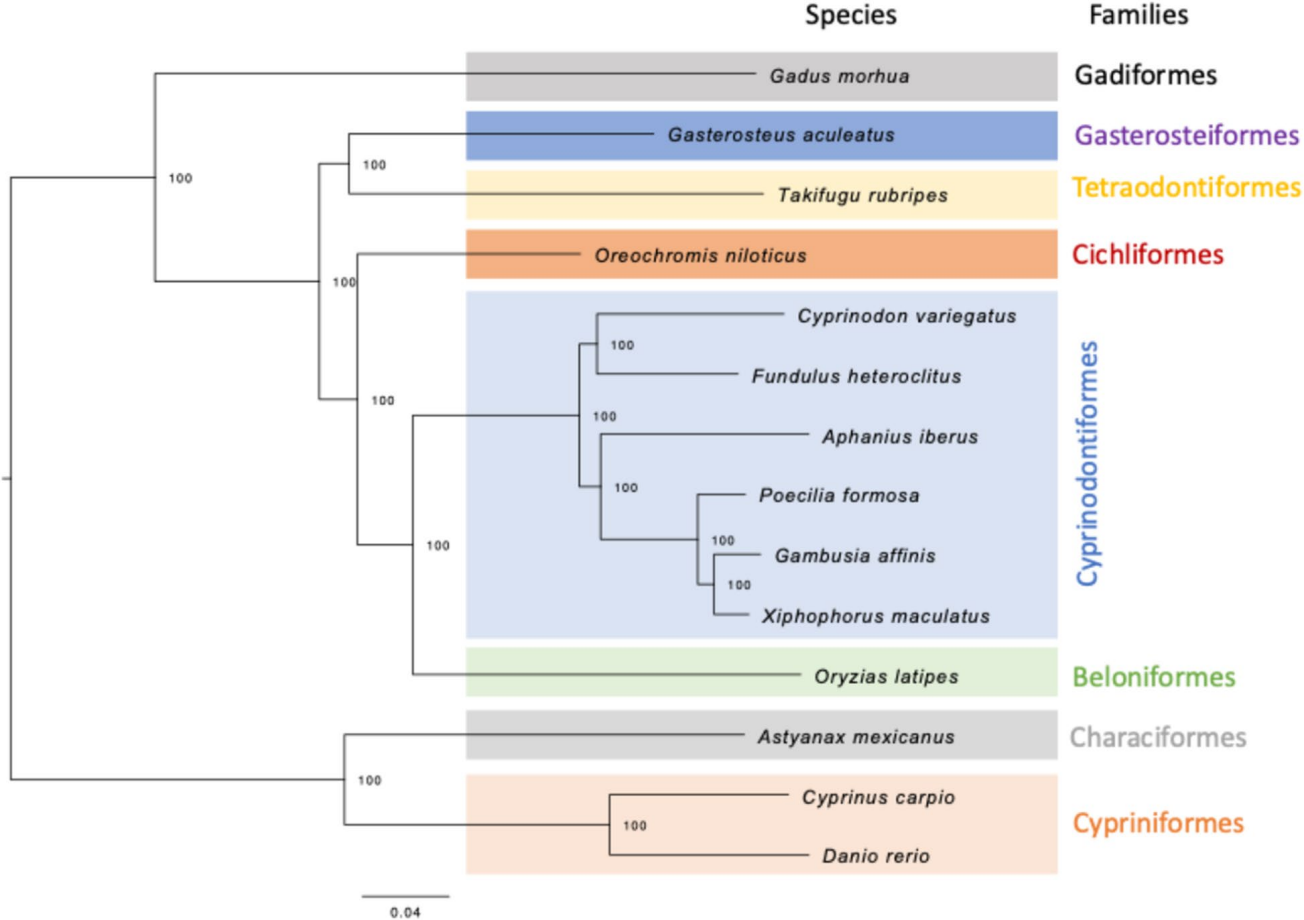
Phylogenetic tree inferred by Orthofinder and IQ-tree. The numbers at nodes represent bootstrap values

The Orthofinder analysis clustered genes from all the 14 species (Table 1) into 25,144 orthogroups, with approximately 97,4% of the total genes assigned to at least one orthogroup. A maximum likelihood phylogenetic tree was constructed with IQ-tree using the database of 2,367,048 amino acids obtained from the Orthofinder analyses (Fig. 6). The tree clustered the order Cyprinodontiformes with the Japanese medaka (*Oryzias latipes*) from the order Beloniformes and the Nile tilapia (*Oreochromis niloticus*) from the order Cichliformes, as the most closely related groups.

**Fig. 7:**
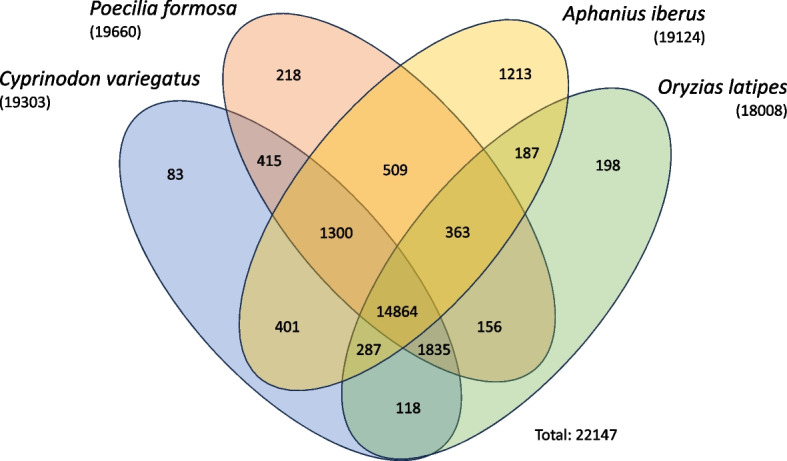
Orthogroups of genes exclusive to and shared among the genome transcripts of the four analyzed fish species: *Aphanius iberus*, *Poecilia formosa*, *Cyprinodon variegatus* and *Oryzias latipes*

The Orthofinder analysis that incorporated only the species, *A. iberus*, *C. variegatus*, *P. formosa* and *O. latipes* showed that of the total number of orthogroups (22,147), approximately 67% (14,864 orthogroups) were shared across all four species. Only about 1% of the orthogroups were found to be species-specific, with 1,213 orthogroups assigned exclusively to *A. iberus,* 83 to *C. variegatus*, 218 to *P. formosa*, and 198 to *O. latipes. A. iberus* shared exclusively 509 orthogroups with *P. formosa*, 401 with *C. variegatus* and 187 with *O. latipes*. Additionally, 1,300 orthogroups were identified as exclusive to the order Cyprinodontiformes (Fig. 7).

## Discussion

**Fig. 8:**
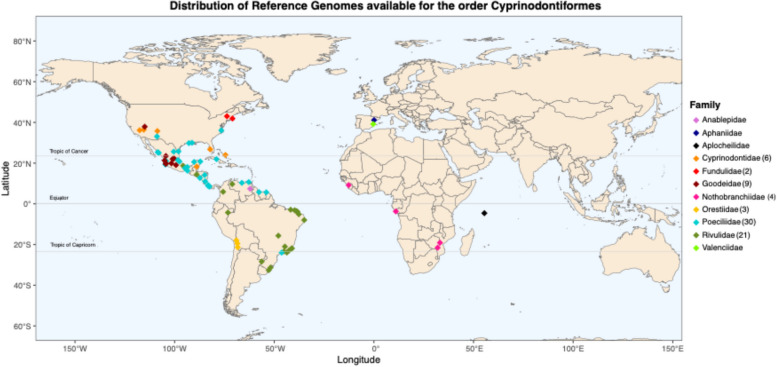
Geographic distribution of Reference Genomes data available in GenBank, color and shape-coded by family. Numbers in brackets on the legend indicate the number of species belonging to each family when they are higher than 1 species per family

The de novo hybrid assembly of the genome of the endangered Spanish toothcarp, *A. iberus*, presented in our study, is of paramount importance as it was the first released Reference Genome of a Euro-Asian species within the order Cyprinodontiformes. The only other such genome released is that of the Valencia toothcarp, *Valencia hispanica* (Fig. 1, Fig. 8). This research provides significant insights into the biological understanding and conservation of threatened species such as euryhaline toothcarps, which have a high potential to adapt to different habitat salinity conditions. This is particularly relevant in light of the ongoing impact of salinization variation and seawater intrusion on the Mediterranean coastal wetlands [95, 96].

While genome sequencing has become a crucial resource in fish genomics research, complete genome sequences remain scarce and unevenly distributed across genera within different orders of fishes as well as throughout their global distribution. This is exemplified by the order Cyprinodontiformes, in which the majority of studies and Reference Genomes have been developed for American species [97–100] (Fig. 8). Out of the 79 Reference Genomes available in GenBank for the order Cyprinodontiformes, which includes more than 1900 species [101], only five families account for 70 of them: (Poeciliidae (30), Rivulidae (21) Nothobranchiidae (4), Goodeidae (9) and Cyprinodontidae (6)). The remaining nine Reference Genomes are distributed sparsely across all other six families. There are four families that currently lack fully sequenced genomes, highlighting the need for more diverse genomic resources (Fig. 8).

The genome assembly was of high quality, as evidenced by a comparative analysis of the genome size, the quality and completeness of the sequencing, and the GC content (39%), when compared with related species. The size of the de novo sequenced genome of *A. iberus* (1.15 Gb) is likely to be similar to that of closely related species, such us *Valencia hispanica*: 1,231.84 Mb (GCA_963556495.1), *Fundulus heteroclitus*: 1,203 Mb (GCA_011125445.2), *Cyprinodon brontotheroides*: 1,163 Mb (GCA_018398635.1), *Girardinichthys multiradiatus:* 1,150 Mb (GCA_021462225.2), or *Anableps anableps*: 867.6 Mb (GCA_014839685.1). The quality and completeness of the genome, assessed using various methods, exceed the minimum thresholds (90%) proposed by a recent study for evaluating genome sequenced data quality [102]. The BUSCO analysis revealed that 92.5% are complete gene copies (Fig. 3). Furthermore, BWA and SAMtools mapped back to the assembly 99.67% of short sequencing reads, while BlobTools v1.1.1 mapped 98.88% (Fig. 2). Additionally, the variation in GC content between the *A. iberus* genome and the genomes of the other species analyzed revealed no sequencing-based GC preferences, indicating the high quality of the genome assembly. In previous studies, it has been proposed that assemblies with a minimum N50 value between 200 kb and 1 Mb should be employed to identify big synteny blocks with an error rate below 5% [103]. The subread Scaffold N50 value of the de novo sequencing genome of *A. iberus* was 1,600 Mb, which is similar to the previous sequenced genomes of other species in the same order, such as *Poecilia formosa* (N50: 1,574 Mb, GCA_000485575.1), *Nothobranchius kuhntae* (N50: 1,178 Mb, GCA_006942095.1), *Aphyosemion austral* (N50: 1,435 Mb, GCA_006937985.1), or *Callopanchax toddi* (N50: 1,656 Mb, GCA_006937965.1). The comparison between the genomes of *Poecilia reticulata* and *A. iberus* revealed a significant degree of sequence similarity, despite differences in their assembly levels (scaffolds *vs.* chromosomes). Collectively, these analyses affirmed the precision, robustness, and reliability of the genome assembly.

High percentage of the *A. iberus* genome was constituted by repetitive elements, a typical pattern observed in eukaryotes which can suggest a strong selective pressure [33, 104, 105]. The Tc1 DNA transposon was observed to be present in the second most common group (Tc1-IS630-Pogo). Previously, Tc1 had been identified as one of the most prevalent Repetitive Element in freshwater bony fish when phylogenetic considerations are not taken into account. Besides, the freshwater environment was observed to be a more favorable environment for the proliferation of the Tc1 transposon [33].

To ensure the reliability of the results and to evaluate the quality of the assembly based on sequence homology (orthogroups), orthology comparisons were conducted with closely related species, including well-studied models (Figs. 6 and 7). The results demonstrated a significant degree of overlap in transcripts across all assemblies, particularly when a more restricted analysis involving four closely related species was performed. The results of the analysis demonstrated a high degree of congruence with phylogenetic relationships among the species, with a minimal percentage of unassigned genes throughout the analysis. Additionally, the number of orthogroups that were exclusive or shared among these four species was found to be similar, highlighting the conserved nature of certain gene groups (Fig. 7). The phylogenetic tree constructed with IQ-tree revealed that *A. iberus*, along with the other members of the order Cyprinodontiformes and closely related genera, was also positioned in accordance with previous studies [106–108] (Fig. 6).

Our study provides further insights by additionally providing the complete genome annotation for this Reference Genome. This information can be used in future studies to identify potential candidate genes involved in the processes of adaptation and resilience to several external abiotic stressors, such as salinity, temperature and hypoxia. In particular, and given the euryhaline condition of *A. iberus*, our annotation has identified over 2,300 genes with a function related to salinity out of the 19,960 genes annotated with Sma3s. particularly hyperosmotic and hypotonic salinity responses, osmosensory signaling, ion transport, ion transmembrane transport, bicellular tight junctions, and hormone systems such as, among others, the renin–angiotensin–aldosterone system. These findings align with similar results in other research, implying that osmoregulation genes are reasonably stable across studies [3]. Nevertheless, more comprehensive comparisons are necessary due to variations in gene annotation methods across datasets. Therefore, the potential for conducting genetic studies on this species, such as its tolerance to varying salinity levels or gene expression responses, among others topics, remains highly intriguing. For instance, it is known that genes involved in osmoregulation play a crucial role for the survival of different Aphaniidae species [109]. These genes mediate differential gene expression in response to varying environmental conditions, enabling the maintenance of internal homeostasis at different salinity levels [109, 110]. A variety of proteins with diverse functions are implicated in these processes, including ion transporters, water channels, barrier proteins, signaling enzymes, and structural components. Regarding claudins and occludins, which are major transmembrane tetraspan proteins of tight junctions, have been described to play an important role in regulating paracellular permeability and ion and molecule equilibrium in several organisms [111, 112]. These two protein families are involved in the gill permeability changes during the process of acclimatization to fluctuating salinity conditions in fishes [113, 114].

Major Intrinsic Proteins (MIPs), including aquaporins and aquaglyceroporins, are essential for osmoregulation. Aquaporins function as water channels vital for hydric and osmotic regulation across cellular membranes, helping maintain homeostasis under stress conditions such as drought and salinity changes [115–120]. Additionally, proteins like the sodium–potassium pump (Na^+^/K^+^ ATPase), NKCC2, and NBCe1 facilitate ion exchange processes, while various proteins with specific functions, such as cathepsins, immunoglobulins, actins, connexins, and GTPases, are expressed in different tissues [121–123].

Our gene annotation has identified several hormone systems crucial for salinity adaptation, including the renin-angiotensin system, which regulates blood pressure and osmoregulation in teleost fishes, favoring cardiovascular homeostasis and renal sodium and water reabsorption [124–128]. Euryhaline species particularly benefit from these osmoregulatory behaviors. Another important hormone linked to osmotic regulation is arginine vasotocin, which is associated with aquaporin function [129–132].

## Conclusions

The hybrid assembly presented in this study represents a significant step forward in our understanding of the biology of *A. iberus*, providing a well-sequenced and annotated Reference Genome that enhances our knowledge of the globally distributed order Cyprinodontiformes, which is currently predominantly limited to species from America and Africa. The application of more recent sequencing technologies, such as Omni-C, Chicago, or Hi-C could further enhance the assembly, achieving chromosomal-level resolution and addressing additional questions in the future.

These findings will contribute to the expanding database of Reference Genomes and will provide valuable information that can facilitate future studies not only in the species but in the whole order. Moreover, the integration of genomic data with predicted genes offers a wide range of research opportunities across various disciplines, including physiology, reproduction, disease, and comparative genomic studies.

## Supplementary Information

Additional file 1.

## Data Availability

The Whole Genome Shotgun project has been deposited at DDBJ/ENA/GenBank under the accession number JAPXFQ01. The Submitted GenBank assembly described in this paper is version GCA_028564705.1. BioProject: PRJNA913687. BioSample: SAMN32303939. Annotation of genes are available at http://hdl.handle.net/10261/365271.
