# Supplementary material for: De novo genome hybrid assembly and annotation of the endangered and euryhaline fish Aphanius iberus (Valenciennes, 1846) with identification of genes potentially involved in salinity adaptation
Source: BMC Genomics. 2025 Feb 12;26:136. doi: 10.1186/s12864-025-11327-0 (PMC11817801; doi:10.1186/s12864-025-11327-0)
Supplement: Supplementary file 1 — Additional file 1. [file 12864_2025_11327_MOESM1_ESM.docx]

**SUPPLEMENTARY MATERIAL**

Supplementary Table 1. Standard metrics for PacBio Sequel II sequence data computed by SequelTools.* PSR and ZOR are the polymerase-to-subread ratio andZMW-occupancy-ratio, respectively

|  | **Nº of reads** | **Nº of bases (bp)** | **Mean read length (bp)** | **N50** |
| --- | --- | --- | --- | --- |
| **Subread** | 7,307,982 | 70,167,158,383bp | 9,601bp | 13,224 |
| **LongestSubread** | 5,452,968 | 50,939,286,495bp | 9,342bp | 13,231 |
| **PSR* = 0.726** |  |  |  |  |
| **ZOR* = 0.746** |  |  |  |  |

Supplementary Table 2. Number of scaffolds and total length (in bp) of the two approaches to genome assembly, both before and after polishing

|  | **MaSuRCA** | **MaSuRCA polished** | **HASLR** | **HASLR polished** |
| --- | --- | --- | --- | --- |
| Contigs (>= 0 bp) | 3026 | 3026 | 8645 | 8645 |
| Contigs (>= 1000 bp) | 2799 | 2799 | 8583 | 8583 |
| Contigs (>= 5000 bp) | 2160 | 2160 | 7986 | 7980 |
| Contigs (>= 10000 bp) | 1979 | 1979 | 7438 | 7427 |
| Contigs (>= 25000 bp) | 1798 | 1798 | 6302 | 6294 |
| Contigs (>= 50000 bp) | 1558 | 1558 | 5036 | 5020 |
|  |  |  |  |  |
| Total length (>= 0 bp) | 1198911485 | 1198861738 | 1137017190 | 1131748719 |
| Total length (>= 1000 bp) | 1198758781 | 1198709059 | 1136981845 | 1131713374 |
| Total length (>= 5000 bp) | 1196877931 | 1196828256 | 1135110062 | 1129824051 |
| Total length (>= 10000 bp) | 1195596068 | 1195546336 | 1131139670 | 1125805715 |
| Total length (>= 25000 bp) | 1192436288 | 1192386761 | 1111739693 | 1106470005 |
| Total length (>= 50000 bp) | 1183690219 | 1183641275 | 1065456247 | 1059915006 |
|  |  |  |  |  |
| Scaffolds | 3026 | 3026 | 8613 | 8613 |
|  |  |  |  |  |
| Largest contig | 9180531 | 9180391 | 1921666 | 1914211 |
| Total length | 1198911485 | 1198861738 | 1137002120 | 1131733649 |
|  |  |  |  |  |
| GC (%) | 39.17 | 39.17 | 39.08 | 39.12 |
|  |  |  |  |  |
| N50 | 1678944 | 1678775 | 285865 | 284695 |
| N75 | 317020 | 317008 | 139428 | 68033 |
| L50 | 201 | 201 | 1180 | 1178 |
| L75 | 834 | 834 | 2607 | 4317 |
|  |  |  |  |  |
| N's per 100 kbp | 0.24 | 0.24 | 0 | 0 |

Supplementary Table 3. Number of masked repeats and their respective length, categorized by repeat class

|  | **Percentage of sequence** | **Number of elements** | **Length occupied** |
| --- | --- | --- | --- |
| **Retroelements** | 14,11 | 400644 | 169164227bp |
| **SINEs:** | 0,41 | 31585 | 4885021bp |
| Penelope | 0,21 | 6971 | 2561018bp |
| **LINEs:** | 11,26 | 297073 | 134957488bp |
| CRE/SLACS | 0 | 0 | 0bp |
| L2/CR1/Rex | 7,62 | 217107 | 91376149bp |
| R1/LOA/Jockey | 0,27 | 9088 | 3291256bp |
| R2/R4/NeSL | 0,19 | 6574 | 2257795bp |
| RTE/Bov-B | 1,21 | 34110 | 14452798bp |
| L1/CIN4 | 1,41 | 17400 | 16908925bp |
| **LTR elements:** | 2,45 | 71986 | 29321718bp |
| BEL/Pao | 0,17 | 1469 | 2090102bp |
| Ty1/Copia | 0,04 | 1009 | 458327bp |
| Gypsy/DIRS1 | 0,74 | 15420 | 8874196bp |
| Retroviral | 0,7 | 8654 | 8408350bp |
| **DNA transposons** | 16,37 | 787189 | 196227672bp |
| hobo-Activator | 5,44 | 277788 | 65272112bp |
| Tc1-IS630-Pogo | 7,22 | 349969 | 86588295bp |
| En-Spm | 0 | 0 | 0bp |
| MULE-MuDR | 0,07 | 3157 | 809587bp |
| PiggyBac | 0,05 | 1523 | 641181bp |
| Tourist/Harbinger | 1,77 | 68169 | 21162212bp |
| Other (Mirage, P-element, Transib) | 0,21 | 8159 | 2482284bp |
| **Rolling-circles** | 0,15 | 4941 | 1785275bp |
| **Unclassified:** | 18,63 | 952460 | 223288827bp |
| **Total interspersed repeat:** | 49,1 |  | 588680726bp |
| **Small RNA:** | 0,27 | 18037 | 3268909bp |
| **Satellites:** | 0,01 | 317 | 112666bp |
| **Simple repeats:** | 0,96 | 235865 | 11563687bp |
| **Low complexity:** | 0,13 | 31217 | 1514348bp |


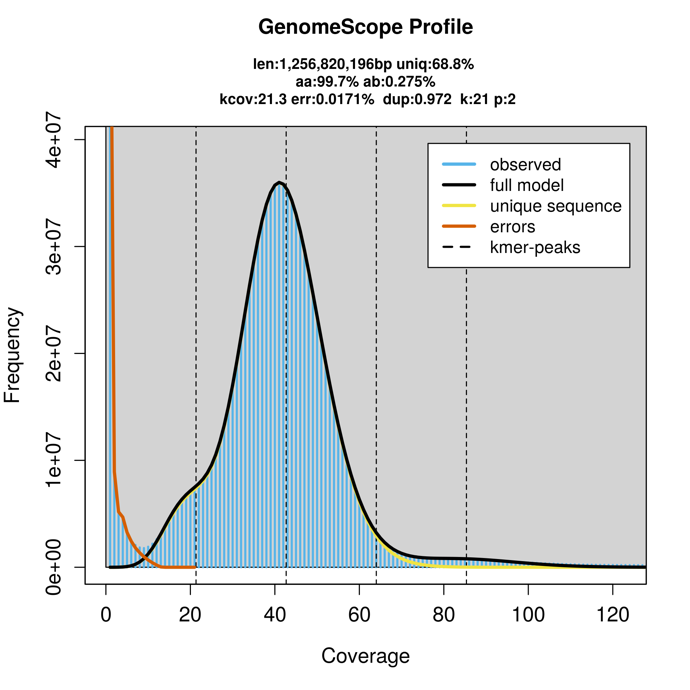


Supplementary Figure 2. The k-mer profile and model fit plot for the genome of A. iberus based on a k-mer size of 21 were generated using GenomeScope. The observed k-mer frequency distribution is shown in blue and represents the coverage and total number of k-mers with a given coverage (frequency)


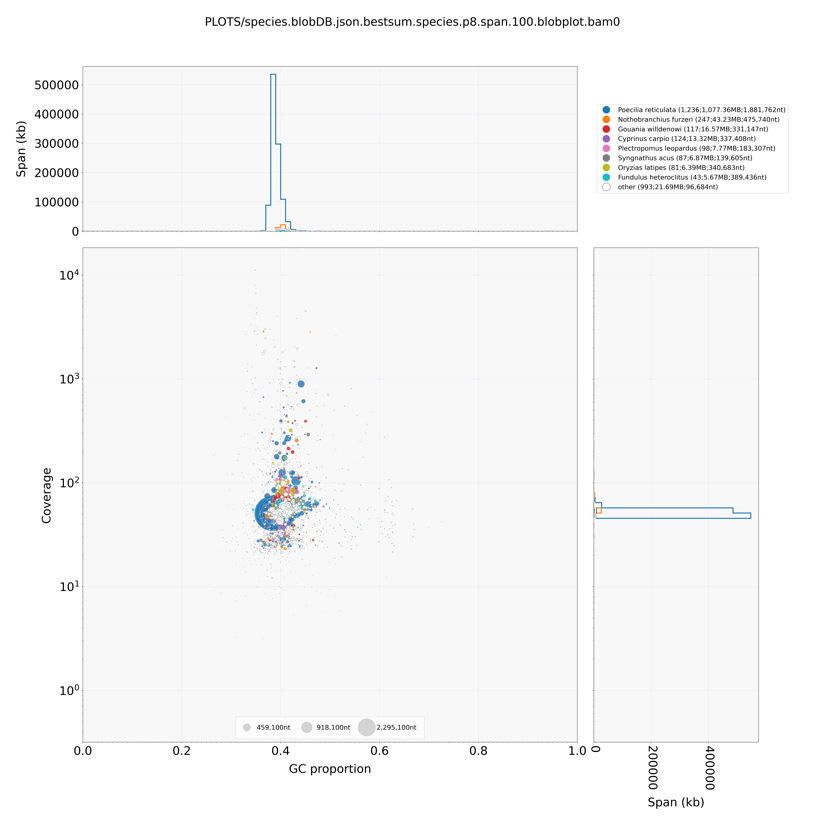


Supplementary Figure 3. Scatter plot representing assembly contigs/scaffolds as dots colored according to taxonomic affiliation, based on sequence similarity search results
